# Intensive mannitol slow infusion post‐stenting may attenuate stenting‐related early adverse effects in patients with cerebral venous sinus stenosis

**DOI:** 10.1111/cns.14350

**Published:** 2023-07-09

**Authors:** Guangyu Han, Shuling Wan, Chaitu Dandu, Da Zhou, Yuchuan Ding, Xunming Ji, Ran Meng

**Affiliations:** ^1^ Department of Neurology, Xuanwu Hospital Capital Medical University Beijing China; ^2^ Advanced Center of Stroke Beijing Institute for Brain Disorders Beijing China; ^3^ National Center for Neurological Disorders, Xuanwu Hospital Capital Medical University Beijing China; ^4^ Department of Neurosurgery Wayne State University School of Medicine Detroit Michigan USA

**Keywords:** adverse effects, cerebral venous sinus stenosis, short‐term slow mannitol infusion, stenting

## Abstract

**Aims:**

To analyze intensive slow mannitol poststenting on attenuating stenting‐related early adverse effects in cerebral venous sinus stenosis (CVSS).

**Methods:**

This real‐world study enrolled subacute or chronic CVSS patients from January 2017 through March 2022 and divided them into DSA only and stenting post‐DSA groups. The later group was subdivided into control (without extra mannitol use) and intensive slow mannitol subgroup (immediate extra mannitol 250–500 mL, 2 mL/min infusion post‐stenting) after signed informed consent. All data were compared.

**Results:**

A total of 95 eligible patients entered into final analysis, in which 37 cases underwent DSA only and 58 cases underwent stenting post‐DSA. Finally, 28 patients were entered into intensive slow mannitol subgroup and 30 in control. Stenting group vs. DSA group, HIT‐6 scores and WBC counts were higher in the former (both *p* < 0.001). Intensive slow mannitol subgroup vs. control on the third day post‐stenting, a statistically significant reductions were noticed in the former on WBC counts (6.19 ± 1.86 × 10^9^/L vs. 9.59 ± 2.05 × 10^9^/L); HIT‐6 scores (degree of headache) (40.00 (38.00–40.00) vs. 49.00 (41.75–55.25)) and brain edema surrounding the stent on CT maps (17.86% vs.96.67%), all *p <* 0.001.

**Conclusions:**

Stenting‐related severe headache, inflammatory biomarkers elevation, and brain edema aggravation can be attenuated by intensive slow mannitol infusion.

## Graphical abstract

Stenting may induce temporarily severe headache, abnormal elevated inflammatory biomarkers, and brain edema aggravation especially in local area surround the stent and even intracranial hemorrhage in the early period post‐stenting. Short‐term extra intensive mannitol slow infusion may effectively attenuate stenting‐related early adverse effects mentioned above especially stenting‐related severe headache.

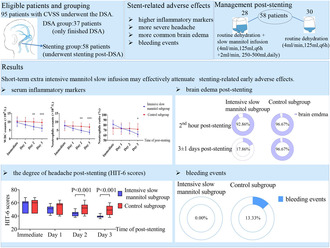

## 1 INTRODUCTION

Cerebral venous sinus stenosis (CVSS) can be due to either cerebral venous sinus thrombosis or non‐thrombotic stenosis. One of the important features of CVSS is intracranial hypertension (IH).^1^, ^2^, ^3^, ^4^, ^5^, ^6^, ^7^, ^8^, ^9^, ^10^, ^11^, ^12^, ^13^, ^14^ Previous studies have identified that venous sinus stenting is an effective method to correct the stenosis. Furthermore, stenting can rectify severe or even fatal IH resulting from CVSS.^1^, ^2^, ^3^, ^4^, ^5^, ^6^, ^7^, ^8^, ^9^, ^10^, ^11^, ^12^ However, stenting‐related early adverse effects, such as severe headache, intracranial hemorrhage, and restenosis are still completely eradicate, which largely deteriorate clinical outcomes.^6^, ^7^, ^9^, ^10^, ^11^, ^15^ In addition, the inflammatory process may cause restenosis of CVSS resulting in poor outcomes.^16^, ^17^

We have noticed that even though the obstruction of venous reflow was corrected, and the mean pressure gradient (MPG) across the stenotic segment was diminished or even disappeared after stenting, patients had even higher serum levels of inflammatory biomarkers and increased brain edema in the initial days post‐stenting compared to their baseline, which may explain why patients with CVSS had even more aggravated symptoms in the early period post‐stenting. Whereby, inhibiting stenting‐related inflammation and brain edema may facilitate clinical outcomes.^18^, ^19^ Herein, we aimed to explore a new strategy to attenuate the early adverse effects of stenting.

## 2 METHODS

### 2.1 Study design and patients' selection

The Ethics Committee of Xuanwu Hospital, Capital Medical University approved this single center real‐world study, and all participates signed the informed consents prior to enrollment. A total of 95 patients with CVSS confirmed by imaging were enrolled from January 2017 through March 2022 consecutively, and all of them underwent digital subtraction angiography (DSA). Medical data were derived from the inpatient database and analyzed by two experienced neurologists and radiologists, respectively. The endovascular interventions were performed by the same experienced surgical team.

Inclusion criteria: (1) the CVSS was confirmed by contrast‐enhanced magnetic resonance venography (CE‐MRV), computed tomographic venography (CTV), or DSA; (2) sub‐acute or chronic CVSS, defined as the interval time from the onset of signs and symptoms until enrollment was ≥4 weeks; (3) persistently or worsening clinical symptoms despite undergoing regular treatment with medications.

Exclusion criteria: (1) a confirmed acute or chronic infection preoperatively; (2) intracranial hypertension (IH) secondary to other reasons, which include (a) drug‐induced IH, (b) cerebrospinal fluid shunt history, (c) intracranial mass occupation, (d) arteriovenous malformations, (e) traumatic brain injury, and (f) acute arterial stroke; (3) and incomplete clinical data or disagreement with the clinical decision making.

### 2.2 Angiography process and venous sinus stenting

All patients with imaging confirmed CVSS further finished DSA with the help of local anesthesia to further identify the degree of the stenosis and to measure the MPG across the stenotic segment. Using the venography and manometry results, the same neurointerventional team stented stenotic segments when the trans‐stenotic MPG was more than 8 mmHg and patients did not have vascular tortuosity, the presence of a long segment lesion, or severe thrombosis in stenotic sinus. Subsequently, the patients were divided into DSA group and stenting group.

### 2.3 Postoperative management in stenting group

**FIGURE 1:**
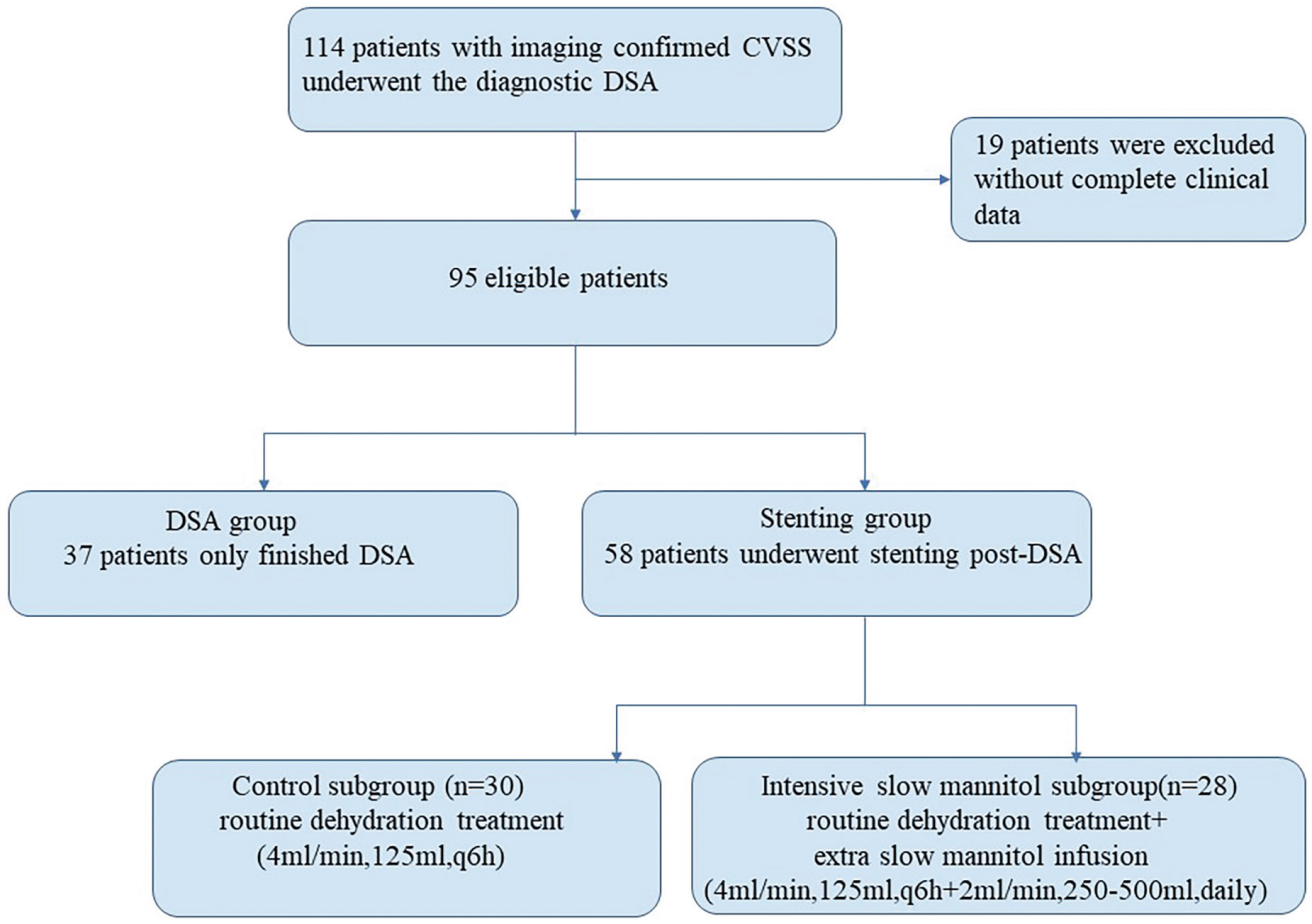
Flow chart of the study. CVSS, cerebral venous sinus stenosis; DSA, digital subtraction angiography.

The routine dehydration strategy for all patients post‐stenting was the same as that prior to stenting (4 mL/min, 125 mL, q6h). Moreover, patients in intensive slow mannitol subgroup underwent an immediate additional slow infusion of mannitol (2 mL/min, 250–500 mL, daily) for 2–3 days after signed the informed consents. Patients who declined to sign the informed consent form were assigned to the control subgroup (Figure 1).

Serum inflammatory biomarkers, including the counts of white blood cell (WBC) and neutrophils, the percentage of neutrophils, interleukin‐6 (IL‐6), and high sensitivity C‐reactive protein (hs‐CRP) and headache intensity (HIT‐6 scores), were assessed at baseline, immediately post‐stenting and on days 1, 2, and 3 post‐stenting. The brain computerized tomography (CT) was assessed at baseline prior to stenting, the immediate post‐stenting, and on post operative day 2.

### 2.4 Observation and quantification of clinical indicators

To access the negative effects of headache on normal activities, the headache intensity was measured by the short‐form Headache Impact Test‐6 (HIT‐6).^20^ Brain edema presented in CT scans was evaluated by a semi‐quantitative approach. A score 0 was assigned to a CT scan if the sulci cerebri in the local area surrounding the stent disappeared completely. If the sulci cerebri could be found in the area, a score of 1 was assigned to the CT scan. Intracranial pressure (ICP) was defined as the opening pressure of the cerebrospinal fluid (CSF) obtained through a lumbar puncture. The trans‐stenotic pressure gradient was represented by MPG measured in DSA.

### 2.5 Statistical analysis

All statistical analyses in this study were conducted by Social Science Statistical Software Package (SPSS) version 28.0 program (IBM). Continuous variables following normal distribution were calculated as mean ± standard deviation (SD) and compared by *t*‐test or two‐way analysis of variance (ANOVA); otherwise, these data were presented as median (interquartile range) and assessed by Mann–Whitney *U* test or non‐parametric Wilcoxon signed‐rank test. Categorical variables were expressed as counts and percentages and Pearson's chi‐square test or Fishers exact test was applied to evaluate differences. Two‐tailed *p* value <0.05 was considered as statistical significance.

## 3 RESULTS

### 3.1 Baseline demographic characteristic

A total of 95 eligible patients were enrolled from January 2017 through March 2022. The mean age was 42 (31–54) years with a mean BMI of 25.95 (23.95–28.72) kg/m^2^. 70.5% of patients identified as females and 29.5% of patients identified as males. A total of 14.7% of patients stated that they consumed alcohol while 13.7% stated they smoked. About 93.7% of patients had chronic CVSS; 60.0% of patients had headache, and 56.8% had visual disorders. About 35 (36.8%) cases reported tinnitus and 25 (26.3%) cases complained of head noises, and 10 (10.5%) cases had hearing disorders. A total of 29.5% of patients were confirmed to have cerebral venous thrombosis (CVT) by imaging. The stenotic segment in cerebral venous sinus was further identified with DSA. The right transverse sinus and right transverse sinus‐sigmoid sinus junction were the most vulnerable sites (30.5% and 23.2%, respectively). Details of the baseline characteristics of the patients are displayed in Table 1.

**TABLE 1:** Baseline demographic data.

Clinical features	All patients, *N* = 95
Demographics	
Age (y), median (IQR)	42 (31–54)
Gender (female), *n* (%)	67 (70.5)
Mean BMI (kg/m^2^), median (IQR)	25.95 (23.95–28.72)
Duration of disease	
Within 4 weeks, *n* (%)	6 (6.3)
More than 4 weeks, *n* (%)	89 (93.7)
Clinical manifestations, *n* (%)	
Headache	57 (60.0)
Visual disorders	54 (56.8)
Tinnitus	35 (36.8)
Head noises	25 (26.3)
Hearing disorders	10 (10.5)
Comorbidities, *n* (%)	
Hypertension	36 (37.9)
Hyperlipidemia	29 (30.5)
Diabetes	9 (9.5)
CVT	28 (29.5)
Life habit, *n* (%)	
Alcohol drinking	14 (14.7)
Smoking	13 (13.7)
Location of venous sinus stenosis, *n* (%)	
Superior sagittal sinus	4 (4.2)
Straight sinus	1 (1.1)
Transverse sinus, *n* (%)	
Right	29 (30.5)
Left	9 (9.5)
Bilateral	5 (5.3)
Sigmoid sinus, *n* (%)	
Right	1 (1.1)
Left	2 (2.1)
Bilateral	1 (1.1)
Lateral sinus, *n* (%)	
Right	6 (6.3)
Left	3 (3.2)
TS‐SS boundary, *n* (%)	
Right	22 (23.2)
Left	5 (5.3)
Bilateral	7 (7.4)

Abbreviations: BMI, body mass index; CVT, cerebral venous thrombosis; TS, transverse sinus; SS, Sigmoid sinus.

About 58 out of the 95 patients underwent stenting post‐DSA (stenting group); the remaining 37 cases underwent DSA only without any stenting (DSA group). The serum inflammatory markers and the results of CT were compared between the two groups immediately following the operation.

In the stenting group, 28 patients into the intensive slow mannitol subgroup and 30 patients in control. Serum inflammatory markers and follow‐up CT results between the two subgroups were compared immediately post‐stenting and within 3 days post‐stenting.

### 3.2 Serum inflammatory biomarkers fluctuation

Complete blood counts, IL‐6 and hs‐CRP in patients were, respectively, tested prior to the operation and immediately after operation with or without stenting.

#### 3.2.1 Pre‐ and post‐DSA

In DSA group, pre‐ vs. post‐DSA: the WBC counts were 5.80 (5.09–6.88) × 10^9^/L vs. 6.75 (5.58–7.91) × 10^9^/L, respectively (*p* < 0.001). Neutrophils counts increased from 3.63 (2.80–4.37) × 10^9^/L to 4.57 (3.93–5.62) × 10^9^/L (*p* < 0.001); and the neutrophils ratio increased from 59.83 ± 9.85% to 72.57 ± 10.85% (*p* < 0.001). The level of IL‐6 was 2.36 (1.76–4.02) mg/L vs. 5.46 (3.83–7.50) mg/L (*p* < 0.001), and the hs‐CRP increased from 1.87 (1.08–3.22) pg/mL to 3.46 (1.86–4.99) pg/mL (*p* < 0.001).

#### 3.2.2 Pre‐ and post‐stenting

In the stenting group, pre‐ vs. post‐stenting: the WBC counts increased from 5.87 (5.09–7.62) × 10^9^/L to 8.66 (7.91–10.18) × 10^9^/L (*p* < 0.001). Neutrophils counts increased from 3.44 (2.82–4.68) × 10^9^/L to 6.17 (5.34–7.88) × 10^9^/L (*p* < 0.001) and the neutrophils ratio increased from 60.39 ± 10.35% to 73.61 ± 12.06% (*p* < 0.001). The level of IL‐6 increased from 2.94 (1.67–5.03) mg/L to 6.48 (4.08–10.07) mg/L (*p* < 0.001), and the level of hs‐CRP increased from 1.41 (0.43–3.64) pg/mL to 4.76 (3.16–6.09) pg/mL, respectively (*p* < 0.001). It is clear that both DSA alone and stenting can increase serum inflammatory biomarkers (Table 2).

**TABLE 2:** Clinical data pre‐ and post‐operation in DSA group and stenting group.

Items	DSA group (*n* = 37)			Stenting group (*n* = 58)		
	Pre	Post	*p* Value	Pre	Post	*p* Value
WBC counts (×10^9^/L)	5.80 (5.09–6.88)	6.75 (5.58–7.91)	<0.001	5.87 (5.09–7.62)	8.66 (7.91–10.18)	<0.001
Neutrophils counts (×10^9^/L)	3.63 (2.80–4.37)	4.57 (3.93–5.62)	<0.001	3.44 (2.82–4.68)	6.17 (5.34–7.88)	<0.001
Neutrophils ratio (%)	59.83 ± 9.85	72.57 ± 10.85	<0.001	60.39 ± 10.35	73.61 ± 12.06	<0.001
IL‐6 (mg/L)	2.36 (1.76–4.02)	5.46 (3.83–7.50)	<0.001	2.94 (1.67–5.03)	6.48 (4.08–10.07)	<0.001
hs‐CRP (pg/mL)	1.87 (1.08–3.22)	3.46 (1.86–4.99)	<0.001	1.41 (0.43–3.64)	4.76 (3.16–6.09)	<0.001
ICP (mmH2O)	–	–	N/A	330.00 (307.50–330.00)	170.00 (155.00–190.00)	<0.001
MPG (mmHg)	–	–	N/A	11.03 (9.56–12.14)	0.00 (0.00–0.74)	<0.001

Abbreviations: DSA, digital subtraction angiography; hs‐CRP, high sensitivity C‐reactive protein; ICP, intracranial pressure; IL‐6, interleukin‐6; MPG, mean pressure gradient; WBC, white blood cell.

#### 3.2.3 Post‐DSA vs. post‐stenting

While both DSA and stenting groups had elevated WBC counts compared to their baseline, the relative elevation of WBC counts in the stenting group was more remarkable: the ratio of WBC counts elevation was 43.24% (22.65%–67.85%) in the stenting group [(post‐stenting–baseline)/baseline], and 8.24% (2.08%–21.25%) in the DSA group [(post‐DSA–baseline)/baseline], *p* < 0.001. The elevated ratios of neutrophils counts and hs‐CRP in the stenting group were also more remarkable than that of the DSA group: stenting group vs. DSA group were 70.00% (43.43%–119.87%) vs. 33.69% (20.37%–50.61%) (*p* < 0.001); and 213.62% (85.96%–613.07%) vs. 83.16% (30.21%–130.32%) (*p* < 0.001), respectively. While, the elevations of both neutrophils ratio and IL‐6 levels between the two groups showed no significant difference [stenting group vs. DSA group were 19.39% (8.26%–34.65%) vs. 19.14% (9.74%–34.33%) (*p* = 0.951); and 99.66% (63.71%–148.97%) vs. 104.57% (55.06%–158.73%) (*p* = 0.994), respectively], details are shown in Table 3.

**TABLE 3:** The ratios of inflammatory markers elevation in DSA group and stenting group [(post‐DSA–baseline)/baseline or (post‐stenting–baseline)/baseline].

Items	DSA group (*n* = 37)	Stenting group (*n* = 58)	*p* Value
WBC counts (%)	8.24 (2.08–21.25)	43.24 (22.65–67.85)	<0.001
Neutrophils counts (%)	33.69 (20.37–50.61)	70.00 (43.43–119.87)	<0.001
Neutrophils ratio (%)	19.14 (9.74–34.33)	19.39 (8.26–34.65)	0.951
IL‐6 (%)	104.57 (55.06–158.73)	99.66 (63.71–148.97)	0.994
hs‐CRP (%)	83.16 (30.21–130.32)	213.62 (85.96–613.07)	<0.001

Abbreviations: DSA, digital subtraction angiography; WBC, white blood cell; IL‐6, interleukin‐6; hs‐CRP, high sensitivity C‐reactive protein.

#### 3.2.4 Intensive mannitol subgroup vs. control subgroup post‐stenting

**FIGURE 2:**
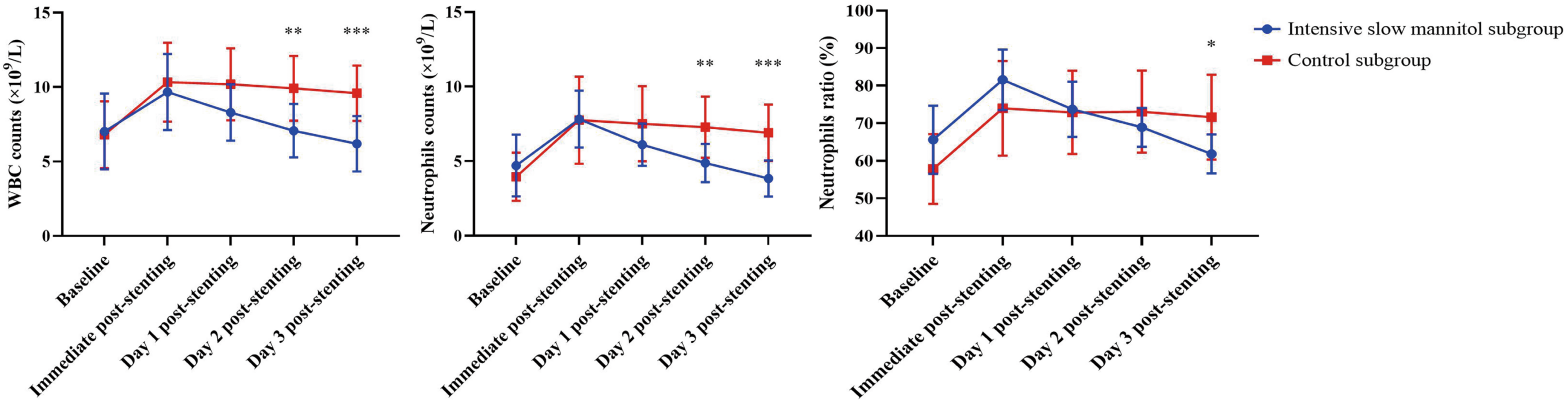
Dynamic fluctuation of serum inflammatory biomarkers post‐stenting in the two stenting subgroups [Two‐way RM ANOVA with Sidak's multiple comparisons test (* versus control subgroup)]. WBC, white blood cell.

Serum inflammatory biomarkers of all patients in the stenting group were monitored dynamically in the initial 3 days post‐stenting. All of the WBC counts, neutrophils counts, and neutrophils ratio in the two subgroups decreased gradually over time. Although a significant difference between the two subgroups was not noticed on day 1 (intensive slow mannitol subgroup vs. control: WBC counts: 8.30 ± 1.89 × 10^9^/L vs. 10.19 ± 2.41 × 10^9^/L (*p* = 0.132); neutrophils counts: 6.10 ± 1.41 × 10^9^/L vs. 7.51 ± 2.40 × 10^9^/L (*p* = 0.319); neutrophils ratio: 73.72 ± 7.36% vs. 72.90 ± 10.62% (*p* = 1.000)), there was a remarkable decrease in WBC and neutrophils counts on day 2 and day 3 (all *p* < 0.01) (Table 5, Figure 2). The WBC and neutrophils counts in the intensive slow mannitol subgroup decreased very rapidly on day 2 (intensive slow mannitol subgroup vs. control: WBC counts: 7.07 ± 1.79 × 10^9^/L vs. 9.92 ± 2.22 × 10^9^/L(*p* = 0.007); neutrophils counts: 4.88 ± 1.28 × 10^9^/L vs. 7.28 ± 2.14 × 10^9^/L (*p* = 0.007)) and day 3 (intensive slow mannitol subgroup vs. control: WBC counts: 6.19 ± 1.86 × 10^9^/L vs. 9.59 ± 2.05 × 10^9^/L (*p* < 0.001); neutrophils counts: 3.84 ± 1.21 × 10^9^/L vs. 6.90 ± 1.96 × 10^9^/L (*p* < 0.001)). The decrease in neutrophils ratio in the slow infusion of mannitol subgroup was not remarkable compared to control subgroup: 68.90 ± 5.16% vs. 73.10 ± 9.84% (*p* = 0.630) on day 2, but 61.83 ± 5.18% vs. 71.62 ± 10.03% (*p* = 0.038) on day 3, respectively (Table 5, Figure 2).

### 3.3 ICP and MPG

Baseline ICP in all patients, represented by the opening pressure of cerebrospinal fluid (CSF), exceeded the normal upper cut‐off levels and had no statistical difference between the DSA group [330.00 (230.00–330.00) mmH2O] and the stenting group [330.00 (307.50–330.00) mmH2O], *p* = 0.073.

Baseline MPG, measured during DSA, was significantly higher in the stenting group [11.03 (9.56–12.14) mmHg] when compared with the DSA group [1.47 (1.10–2.21) mmHg], *p* < 0.001 (Table 4). In the stenting group, the ICP decreased from exceeding 330.00 (307.50–330.00) mmH2O pre‐stenting to 170.00 (155.00–190.00) mmH2O post‐stenting (*p* < 0.001); and the MPG pre‐ and post‐stenting were 11.03 (9.56–12.14) mmHg and 0.00 (0.00–0.74) mmHg, respectively, *p* < 0.001 (Table 2).

**TABLE 4:** Baseline ICP and MPG, postoperative HIT‐6 scores and CT follow up.

Items	DSA group (*n* = 37)	Stenting group (*n* = 58)	*p* Value
Baseline ICP (mmH2O)	330.00 (230.00–330.00)	330.00 (307.50–330.00)	0.073
Baseline MPG (mmHg)	1.47 (1.10–2.21)	11.03 (9.56–12.14)	<0.001
Postoperative HIT‐6 scores			
Immediate	40.00 (40.00–40.00)	60.00 (44.00–62.00)	<0.001
Day 1	38.00 (36.00–39.00)	53.00 (42.75–57.00)	<0.001
Day 2	36.00 (36.00–38.00)	45.00 (42.00–52.00)	<0.001
Day 3	36.00 (36.00–36.00)	41.00 (40.00–50.50)	<0.001
Postoperative brain edema on CT scan, *n* (%)	2 (5.41)	55 (94.83)	<0.001

Abbreviations: ICP, intracranial pressure; MPG, mean pressure gradient; HIT‐6, short‐form Headache Impact Test; CT, computed tomography; DSA, digital subtraction angiography.

### 3.4 HIT‐6 scores and CT scan

We have observed that headache is the most common postoperative complaint after DSA with or without stenting. All patients underwent HIT‐6 evaluation dynamically and CT scan immediately post‐DSA or post‐stenting.

Headache was more severe in the stenting group compared to the DSA group. HIT‐6 scores between the DSA group and stenting group were 40.00 (40.00–40.00) vs. 60.00 (44.00–62.00) immediately after the procedure; 38.00 (36.00–39.00) vs. 53.00 (42.75–57.00) on day 1; 36.00 (36.00–38.00) vs. 45.00 (42.00–52.00) on day 2; and 36.00 (36.00–36.00) vs. 41.00 (40.00–50.50) on day 3, respectively, all *p* < 0.001 (Table 4).

**FIGURE 3:**
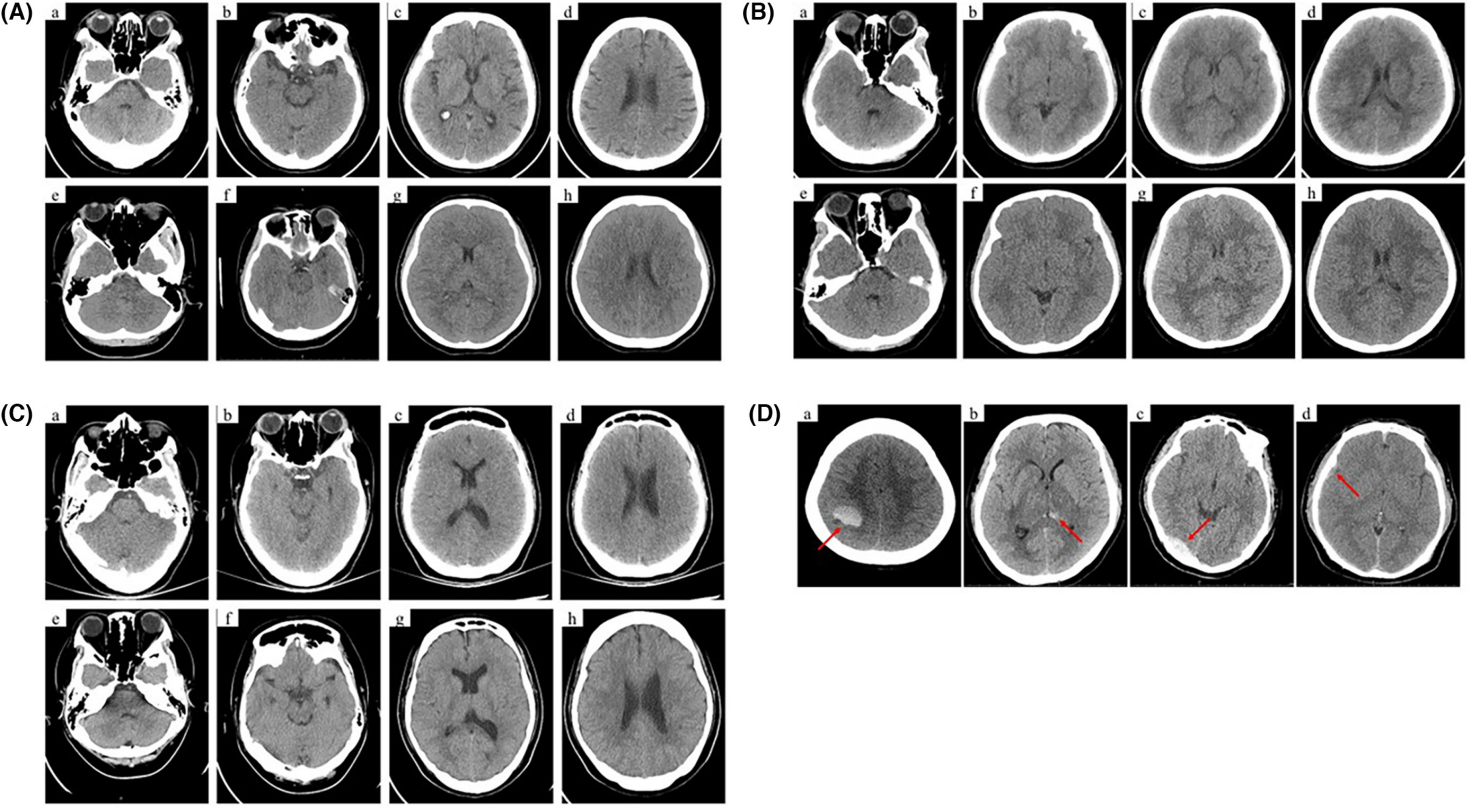
(A) The CT scanned immediate post‐DSA or post‐stenting. (a–d) CT scanned immediate post‐DSA; (e–h) CT scanned immediate post‐stenting. (B) Follow‐up CT of the patient in control subgroup. (a–d) Immediate post‐stenting. (e–h) At Day 2 after stenting. (C) Follow‐up CT of the patient in intensive slow mannitol subgroup. (a–d) Immediate post‐stenting. (e–h) At Day 2 after stenting. (D) Intracranial hemorrhage post‐stenting on CT maps (red arrows): (a) Right parietal lobe hemorrhage; (b) Hemorrhagic transformation within infarcted area; (c, d) Subdural hemorrhage.

Brain edema was present on the CT scan in two out of 37 patients in the DSA group and 55 out of 58 patients in the stenting group immediately after the operation, with 0 score due to the absolute disappeared sulci cerebri in local area surrounding the stent, *p* < 0.001 (Table 4, Figure 3A).

### 3.5 Intensive slow mannitol subgroup versus control post‐stenting

#### 3.5.1 Brain edema in local area surroundingthe stent, HIT‐6 scores and the duration of headache continued

Patients in the stenting group were further subdivided into an intensive slow mannitol subgroup and a control subgroup. Following the CT scan post‐stenting, brain edema was noticed in almost all patients in both subgroups immediate post‐stenting.

In the following days, all patients underwent CT scans of their brain every 1 or 2 days until discharge. The degree of brain edema dissipation, especially those with a score of 0, in the two subgroups was remarkably different. In the control subgroup, 29 patients (96.67%) had brain edema in the local area surrounding the stent, which remained present from 4 to 5 days post stenting and, in some cases, until the seventh day prior to discharge; in the intensive slow mannitol subgroup, the brain edema disappeared within 2 days in a majority of patients with only five patients (17.86%) continuing to have brain edema in the local area surrounding the stent on the third day post‐stenting, *p* < 0.001 (Table 5, Figure 3B,C).

**TABLE 5:** The fluctuation of the inflammatory biomarkers in plasma and the HIT‐6 scores, the duration of headache, the follow‐up CT, bleeding events and inpatient time post‐stenting.

Items	Intensive slow mannitol subgroup (*n* = 28)	Control subgroup (*n* = 30)	*p* Value
The WBC counts post‐stenting (×10^9^/L)			
Day 1	8.30 ± 1.89	10.19 ± 2.41	0.132^a^
Day 2	7.07 ± 1.79	9.92 ± 2.22	0.007^a^
Day 3	6.19 ± 1.86	9.59 ± 2.05	<0.001^a^
The neutrophils counts post‐stenting(×10^9^/L)			
Day 1	6.10 ± 1.41	7.51 ± 2.40	0.319^a^
Day 2	4.88 ± 1.28	7.28 ± 2.14	0.007^a^
Day 3	3.84 ± 1.21	6.90 ± 1.96	<0.001^a^
The neutrophils ratio post‐stenting (%)			
Day 1	73.72 ± 7.36	72.90 ± 10.62	1.000^a^
Day 2	68.90 ± 5.16	73.10 ± 9.84	0.630^a^
Day 3	61.83 ± 5.18	71.62 ± 10.03	0.038^a^

HIT‐6 scores post‐stenting			
Immediate	60.00 (44.00–62.00)	60.00 (44.00–64.00)	0.769
Day 1	51.00 (44.00–54.00)	57.00 (42.00–59.00)	0.066
Day 2	44.00 (40.00–46.00)	52.00 (43.00–57.00)	<0.001
Day 3	40.00 (38.00–40.00)	49.00 (41.75–55.25)	<0.001
The duration of post‐stenting headache (days)	2.50 (0.25–4.00)	4.00 (3.00–6.00)	<0.001
The brain edema in local area surrounding the stent on CT maps on 3rd day post‐stenting, *n* (%)	5 (17.86)	29 (96.67)	<0.001
The bleeding events post‐stenting, *n* (%)	0 (0.00)	4 (13.33)	0.113
Inpatient time after stenting (days)	4.50 (3.00–6.00)	7.00 (6.00–9.00)	<0.001

Abbreviations: HIT‐6, short‐form Headache Impact Test; CT, computed tomography; WBC, white blood cell.

^a^ Means Two‐way ANOVA analysis.

**FIGURE 4:**
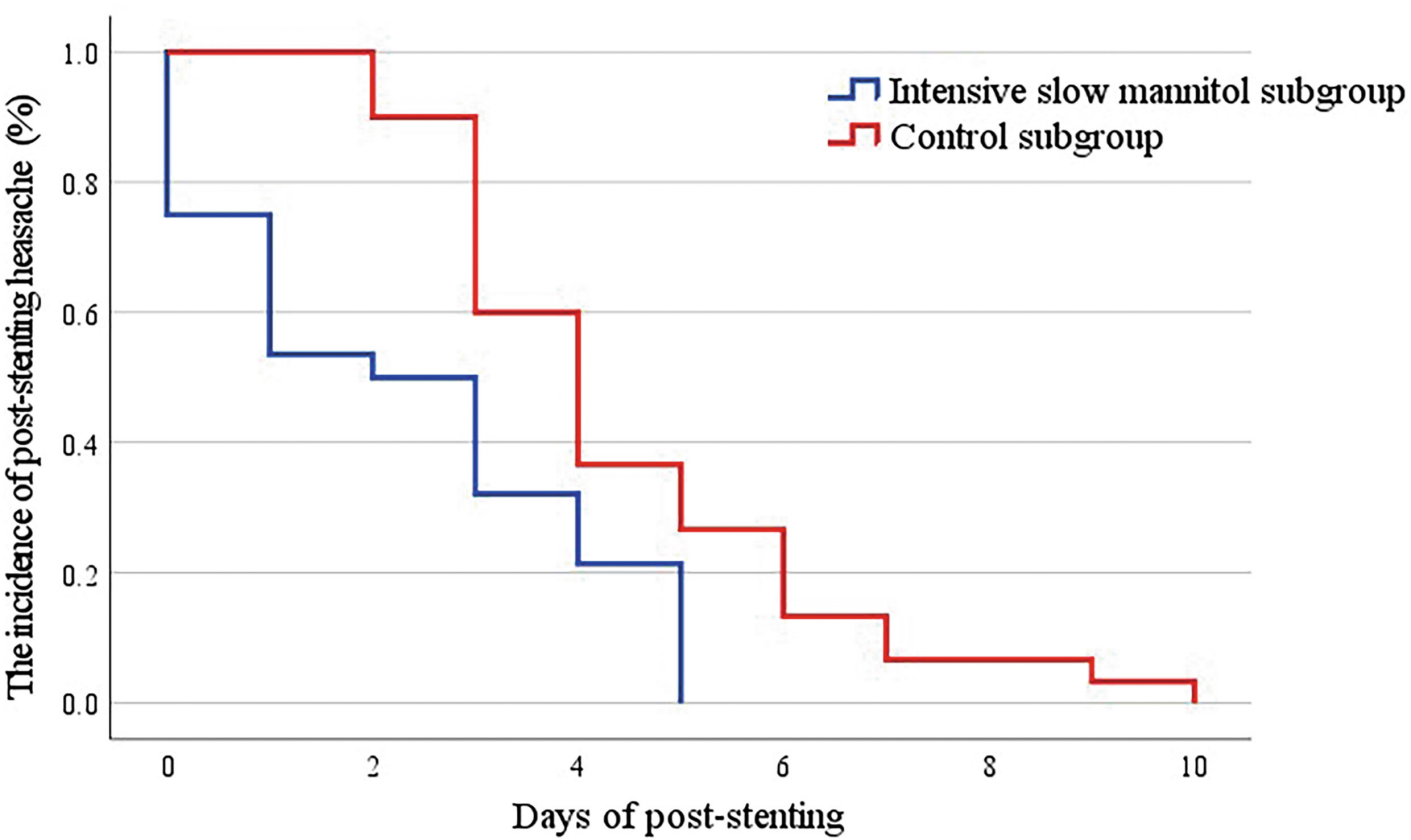
Kaplan–Meier curve of the headache post‐stenting in two stenting subgroups (log‐rank test, *p* < 0.001).

The degree of headache in the two subgroups was very different. In the intensive slow mannitol subgroup vs. control subgroup, HIT‐6 scores were 60.00 (44.00–62.00) vs. 60.00 (44.00–64.00) immediately post‐stenting (*p* = 0.769); 51.00 (44.00–54.00) vs. 57.00 (42.00–59.00) on day 1 (*p* = 0.066); 44.00 (40.00–46.00) vs. 52.00 (43.00–57.00) on day 2 (*p* < 0.001) and 40.00 (38.00–40.00) vs. 49.00 (41.75–55.25) on day 3 (*p* < 0.001). The duration of the headache after stenting was 2.50 (0.25–4.00) days in the intensive slow mannitol subgroup vs. 4.00 (3.00–6.00) days in control subgroup (*p* < 0.001) (Table 5, Figure 4).

#### 3.5.2 Bleeding events and inpatient duration post‐stenting

In this study, no bleeding events occurred in the intensive slow mannitol subgroup (0.00%), while four patients (13.33%) experienced bleeding in the control subgroup post‐stenting. One patient had a hematoma located at right parietal lobe, another patient experienced hemorrhagic transformation inside the area of CVT induced infarction, and two patients had subdural hemorrhages (Figure 3D), details are shown in Table 5. Second, both the elevated serum inflammatory biomarkers and brain edema decreased more rapidly in the intensive slow mannitol subgroup compared to the control subgroup (Table 5, Figures 2 and 3C). Finally, the percentage, severity (HIT‐6 scores), and the duration of headache after intensive slow mannitol infusion post‐stenting were better than that in the control subgroup. All of these factors reduced the time of hospital stay in the intensive slow mannitol subgroup [4.50 (3.00–6.00) days] compared to the control subgroup [7.00 (6.00–9.00) days] (*p* < 0.001), details are displayed in Table 5.

## 4 DISCUSSION

### 4.1 CVSS related IH and treatment

It is well‐known that the etiologies of CVSS include thrombotic and non‐thrombotic causes, the former comes from incomplete recanalization after thrombosis, although the patients have undergone standardized anticoagulation with enough duration.^7^ Non‐thrombotic CVSS mainly occurs due to giant arachnoid granule obstruction in cerebral venous sinus, lesions of cerebral venous sinus wall, or both.^21^ One of the most fatal complications of either type of CVSS is severe IH, resulting in a series of nervous dysfunction, such as paralysis of optic nerve and abducent nerve leading to permanent irreversible visual impairment, visual field defect and diplopia, brain hernia, or even death.^1^, ^2^, ^3^, ^7^, ^9^, ^10^, ^11^ The underlying mechanism may be due to venous sinus outflow obstruction at the stenotic segment, which induces chronic cerebral venous hypertension and prohibits the reabsorption of CSF. The elevated pressure derived from increased levels of CSF compresses brain parenchyma leading to clinical manifestations of IH. Furthermore, because the venous tunica media does not contain many elastic and muscular fibers, external compression from elevated intra cerebral pressure (ICP) can worsen venous stenosis and further obstruct both venous outflow and CSF reabsorption, which then elevates ICP and forms a positive feedback circle.^2^, ^4^, ^5^, ^6^, ^7^, ^9^, ^10^, ^11^, ^17^, ^22^, ^23^

Intracranial venous sinus stenting is considered as a feasible option to correct the stenosis and decrease ICP in CVSS; it has been demonstrated as a safe and effective treatment for patients diagnosed with CVSS‐induced IH.^1^, ^2^, ^3^, ^4^, ^5^, ^6^, ^7^, ^8^, ^9^, ^10^, ^11^ Previous studies, including our study, had identified that stenting, which allowed for brisk flow in involved venous sinuses, could be used to correct CVSS with worsening clinical symptoms or unresponsive to conventional standard anticoagulation.^7^, ^12^, ^13^, ^14^ Additionally, it has been demonstrated that stenting is also a safe and effective method for non‐thrombotic CVSS–induced IH.^1^, ^2^, ^3^, ^4^, ^5^, ^6^, ^8^, ^9^, ^10^, ^11^

However, stenting on CVSS correction is not without limitations. Stenting related severe headache, brain edema, and even bleeding events that seriously affected clinical outcomes and prolong their hospital stay often occurred in clinical setting, which remains an urgent entity that needs to be solved at present.

### 4.2 Endovascular interventions induce inflammation

During the endovascular intervention processes, vascular endothelial injuries induced by the crawling of guide wire catheter in the vascular lumen, contrast injection, and stent placement are probable mechanisms that induce or augment the complex inflammatory responses. Moreover, the hyper‐perfusion resulting from sudden recanalization post‐stenting may also take part in inflammatory processes.^24^, ^25^, ^26^ Whereby, it is expected that serum inflammatory biomarkers will significantly increase after endovascular interventions, which is consistent with our research.^24^, ^26^, ^27^

### 4.3 The probable mechanisms of brain edema post‐stenting

Several studies have concluded that patients with CVSS are prone to elevated ICP and trans‐stenotic pressure gradient through manometry.^1^, ^2^, ^3^, ^4^, ^5^, ^6^, ^7^, ^9^, ^10^, ^11^ Growing evidence has also supported that there are significant decreases in ICP and mean pressure gradient (MPG) across the stenotic segment immediately after stenting.^1^, ^2^, ^3^, ^4^, ^5^, ^6^, ^7^, ^9^, ^10^, ^11^, ^28^ These conclusions are in line with our observations. However, the brain edema, especially in the local area surrounding the stent, presented on follow‐up CTs in almost all patients post‐stenting seems not to conform with the decreased ICP and MPG. The definite mechanism underlying this edema remains unclear. In our observations, baseline ICP in both DSA group and stenting group were both higher than the normal cutoff value with no statistical difference between them. Therefore, it is possible that the causes of brain edema were mainly related to stent implantation rather than baseline elevated ICPs. It is well known that stenting in venous sinus stenosis is different than that in arterial disease. Arterial diseases have a normal ICP environment, whereas venous sinus stenosis induces a high‐pressure environment in the whole brain. When the stent is released successfully, the intracranial pressure of both the local segment and entire brain drops suddenly, causing the local brain tissue to be pulled and the whole brain tissue to suddenly decompress; this may be the main reason for early brain edema after stenting. The possible mechanism is similar to decompression craniectomy. After patients undergo decompressive craniectomy, a significant reduction in ICP has been observed, but brain edema formation is enhanced,^29^, ^30^, ^31^ which may be related to the asymmetric response of the brain parenchyma to decreased ICP throughout the open cranium.^29^ The hyperperfusion of the local decompressed area and the increased tissue pressure gradient of the whole brain result in the development of brain edema.^30^ In addition, the impairment of cerebral autoregulation, failure of cerebral energy metabolism, and increased cerebral inflammation after craniectomy can also contribute to aggravation of brain edema.^31^ Therefore, routine dehydration therapy post‐stenting should not only be continued, but also used more intensively.

### 4.4 Headache and intracranial hemorrhage post‐stenting

There has been numerous research analyzing the adverse effects related to stenting, and the first diagnostic criteria for post‐stenting headache has been proposed in the International Classification of Headache Disorders (3rd edition).^7^, ^32^, ^33^

Our study also observed that stenting‐related headache could either be a considerable worsening of a prior headache or a new onset headache after stenting.

The mechanisms of stenting‐related headache remain unclear. It may be correlated with stent implantation leading to stretching of the venous sinus wall to stimulate intracranial pain‐sensitive structures.^7^, ^33^ In addition, the brain edema, especially in the local area surrounding the stent, found in our study could be also a plausible reasoning for the headache in the early postoperative period after stenting.

There has been increasing attention towards cerebral bleeding post‐stenting, such as cerebral or subdural hematoma and subarachnoid hemorrhage.^6^, ^7^, ^9^, ^10^, ^28^, ^34^ For patients with residual thrombus in the stenotic sinus, the thrombus disruption after stenting might promote activation of local inflammation and coagulation dysfunction, contributing to endothelial cell injuries. This can result in a friable venous wall that is prone to damage and hemorrhage from endovascular maneuvers.^7^, ^35^, ^36^ Moreover, our previous study demonstrated that hemorrhage usually occurred in the brain region contralateral to the side of the stent due to transient pressure differences between the two sides that leads to the contralateral perforating vein being pulled and injured.^15^

In the present study, 4 patients suffered bleeding events in the control subgroup. The bleeding events included a hemorrhage in right parietal lobe, subdural hemorrhages in the right pare, and intracranial hemorrhage in the preoperative infarcted lesion. It is important to optimize treatment strategies after venous sinus stenting to avoid these complications.

### 4.5 Significance of intensive slow mannitol use post‐stenting

Mannitol is considered a mainstay therapy to alleviate cerebral edema and decrease ICP.^37^, ^38^, ^39^ It exerts influence on blood viscosity by increasing plasma osmolality and pulling water intravascularly from tissue.^37^, ^38^ In addition to, mannitol may decrease the cerebrospinal fluid (CSF) formation rate to reduce the ICP.^37^ Apart from the salutary effect on brain swelling and ICP, mannitol is also highly valid for quelling the inflammatory reactions in brain injury and may be cytoprotective through scavenging free radicals released during the inflammation process.^38^, ^39^ In this study, all patients underwent continuous mannitol therapy pre‐stenting, and on this basis, patients in the intensive mannitol subgroup underwent extra mannitol slow infusion post‐stenting. We found for the first time that extra slow infusion of mannitol could further alleviate brain edema prominently and rapidly than a routine mannitol strategy. Moreover, this therapy could also rapidly decrease the levels of abnormal elevated serum inflammatory biomarkers, which effectively attenuates stenting‐related severe headache and may even inhibit the onset of cerebral bleeding events. The probable mechanism may be slow infusion of mannitol prolongs its retention time in the blood circulation, which dehydrates swollen vascular endothelial cells, inhibits inflammatory reactions, and scavenges free radicals. Furthermore, slow infusion of mannitol has weak dehydration effects on brain tissue, so it has little impact on ICP and thus avoids the low intracranial pressure headache caused by excessive rapid dehydration.

### 4.6 Long‐term adverse effects and outcomes post‐stenting

Although stenting‐related inflammatory process is transient, the release of reactive oxygen species and/or cytokines from inflammatory cells contribute to cell proliferation and vascular remodeling, and further increase the risk for restenosis within or adjacent to the stent. Furthermore, the injured endothelial cells post‐stenting lead to platelet adherence, aggregation and activation, which promote the coagulation cascade by inducing thrombin formation. Subsequently, thrombin formation causes thrombosis within or nearby the stent leading to sinus restenosis for patients with intracranial venous stenting.^3^, ^7^, ^25^, ^27^, ^28^, ^33^, ^35^, ^36^ However, this study mainly compared the early adverse effects of post‐stenting prior to discharge between the intensive slow mannitol subgroup and the control subgroup. Follow‐up for long‐term adverse effects and clinical outcomes are still ongoing for these patients.

## 5 LIMITATIONS

Limitations of this study may as follows: a single‐center real‐world study. There is a need to conduct a greater prospective multicenter study in the future. Second, CT follow‐up in stenting group was monitored dynamically prior to discharge without identical cut‐off points, as such the lack of consistent cut off times may act as a confounding factor when it comes to brain edema. Third, the pathological mechanism of brain edema, especially in areas surrounding the stent, is still unclear, and there is no published or unified agreement on methods to assess brain edema. Finally, long‐term outcomes following slow infusion of mannitol post‐stenting are still ongoing.

## 6 CONCLUSIONS

Stenting‐related early severe headache, inflammatory biomarkers elevation, and brain edema aggravation can be attenuated by intensive slowly mannitol infusion.

## AUTHOR CONTRIBUTIONS

RM: manuscript drafting and revision, and study concept and design. GH: manuscript drafting, interpretation of the data, and figure drawing. RM and GH: manuscript writing and final approval of the manuscript. SW, CD, DZ and XJ: data collection. RM and YD: Manuscript drafting and revision.

## FUNDING INFORMATION

This work was supported by the Beijing Natural Science Foundation (7212047) and the National Natural Science Foundation Grants (82171297);

## CONFLICT OF INTEREST STATEMENT

All authors reported no conflicts of interest.

## Data Availability

The datasets generated during this study are available from the corresponding author upon reasonable request.
